# Carcass characteristics of lambs fed diets with increasing levels of crude glycerin

**DOI:** 10.5713/ajas.18.0825

**Published:** 2019-02-14

**Authors:** Caio Alves da Costa, Francisco Fernando Ramos de Carvalho, Adriana Guim, Gilcifran Prestes de Andrade, Daniel Barros Cardoso, Michel do Vale Maciel, Gabriela Gonçalves da Silva, Andreza Guedes de Oliveira Nascimento

**Affiliations:** 1 Department of Animal Science, Federal University of Roraima, UFRR, Boa Vista, 69310-000, Roraima, Brazil; 2 Department of Animal Science, Federal Rural University of Pernambuco, UFRPE, Recife, 52171-900, Pernambuco, Brazil; 3 Department of Animal Science, Federal University of Amazonas, UFAM, Parintins, 69152240 Amazonas, Brazil; 4 Department of Veterinary Medicine, Federal Rural University of Pernambuco, UFRPE, Recife, 52171-900, Pernambuco, Brazil

**Keywords:** Glycerol, Energy, Performance, Sheep, Meat

## Abstract

**Objective:**

An experiment was conducted to evaluate the effects of increasing levels of crude glycerin (0%, 6%, 12%, and 18%) used as a substitute for corn in lamb feed on the quantitative characteristics of the carcass.

**Methods:**

A total of 40 crossbred Santa Inês lambs that were four months old with a mean initial weight of 21.0±0.8 kg were randomly distributed in four treatments with ten replicates. The animals were slaughtered after 66 days of confinement. The effects of crude glycerin as a replacement for corn in the diet of the lambs on the carcass characteristics, commercial cut weight and yield and carcass measurements were studied.

**Results:**

There was an increasing linear effect for body weight at slaughter with the replacement of corn by crude glycerin. The dry matter and metabolizable energy intakes, weight of the empty body, hot carcass weight and cold carcass weight showed a quadratic effect, with maximum crude glycerin levels estimated at 10.9%, 9.8%, 10.83%, 11.78%, and 11.35%, respectively. The initial pH was not influenced by the replacement of corn for crude glycerin, while the final pH presented a quadratic effect. The other parameters of the carcass and the weights and yields of commercial cuts were not influenced. There was also no effect of the diets on carcass morphometric measurements, except for the thoracic perimeter and the carcass compactness index, which presented quadratic and linear effects, respectively.

**Conclusion:**

Crude glycerin can replace up to 18% of corn because it favours muscle tissue deposition without promoting changes in the main carcass characteristics of lambs.

## INTRODUCTION

Among the various agro-industrial by-products currently used in ruminant feed, the most important are those derived from biodiesel production. Glycerin (C_3_H_8_O_3_) is the main co-product generated in the production of biodiesel and approximately 10% of the total volume of biodiesel produced corresponds to glycerin [1]. This co-product results from the formation of methyl esters of fatty acids from triglycerides [2].

Given that feed is the largest cost in livestock production, the use of alternative foods as co-products of biodiesel can be a viable alternative in economic and nutritional terms. Glycerin can be used as an energy ingredient in feed, replacing grains that are more expensive, to increase the profitability of the activity [3].

Glycerol is absorbed by the ruminal epithelium, metabolized in the liver and directed to gluconeogenesis by the action of the enzyme glycerol kinase, which converts it to glucose. Part of glycerol can be fermented to propionate in the rumen, which in turn is metabolized to oxaloacetate via the Krebs cycle in the liver and can be used to form glucose by the gluconeogenic route. Thus, crude glycerine (CG) has a potential application as a gluconeogenic substrate for ruminants [4]. In addition to serving as a source of energy, glycerol may also have positive effects on amino acid retention by inhibiting the activity of the enzymes phosphoenolpyruvate carboxykinase and glutamate dehydrogenase, which results in saving glucogenic amino acids, favouring the deposition of body protein [5].

Diet directly influences the consumption and digestibility of nutrients and consequently, the performance of animals, as well as body and carcass composition. The valuation of the carcass depends on the relationship between body weight and age at slaughter, among other factors. The goal is to obtain higher weights at younger ages to meet consumer market demands [6]. Some studies have evaluated the effects of CG on the diets of sheep and cattle by substituting concentrated feeds and/or associated with urea [7–9], and improvements were achieved or these changes did not impair weight gain and carcass characteristics.

Thus, the objective of this study was to evaluate the replacement of corn with CG in lamb feed on the quantitative and qualitative characteristics of the carcass.

## MATERIALS AND METHODS

### Animal care

The experiment was carried out at the Animal Evaluation Center with Small Ruminants III of the Animal Science Department of the Federal Rural University of Pernambuco (UFRPE), Recife - PE, Brazil. The experimental protocols were approved by the Institutional Animal Care and Use Committee of the Federal Rural University of Pernambuco (CEUA-UFRPE) under license number 059/2016.

### Animals, experimental design and experimental diets

We used 48 Santa Ines non-castrated male lambs that were four months old and had an average initial body weight of 21.0±0.8 kg. Initially, lambs were identified, de-wormed with Ivermectin, immunized against clostridia and supplemented with an ADE vitamin compound. The animals were confined in individual pens with feeders and water suppliers. Initially, lambs were submitted to a period of 24 days to adapt to the experimental diets. After the adaptation period, the animals were randomly assigned one of four experimental treatments, which consisted of four concentrations of CG (0%, 6%, 12%, and 18% of dry matter [DM]) as a substitute for ground corn, with ten replicates per treatment.

The ingredients used were tifton hay, ground corn, CG, soybean meal, urea, ammonium sulfate, mineral supply, limestone calcitic and dicalcium phosphate (Table 1). Crude glycerine was obtained from the production of biodiesel from cottonseed oil at the Bioenergy Unit of the Center for Strategic Technologies of the Northeast, Caetés (PE), Brazil. The diets were formulated with the aim of an average daily gain of 250 g, according to the nutritional recommendations of the National Research Council [10] (Table 2).

### Experimental procedures and sampling

The diets were provided as a complete mixture in individual feeders twice daily at 08:00 (50%) and 16:00 (50%), allowing 15% leftovers. Weekly samples were collected from the supplied diets (offer and refusal), and then the ingredients and leftovers were pre-dried in a forced ventilation oven at 55°C for 72 hours and ground in a Willey type mill with a 1 mm sieve. They were then submitted to further chemical analysis for their content of DM, organic matter, crude protein (CP), and ethereal extract (EE) which was determined according to the AOAC [11] with methods numbers 967.03, 942.05, 981.10, and 920.29, respectively. Neutral detergent fibre (NDF) was determined according to Mertens [12], with corrections for protein and ash according to the methodologies described by Licitra et al [13] and Mertens [12], respectively. The concentrations of glycerol, methanol and sodium of the CG were determined by gas chromatography.

The total digestible nutrients (TDN) was estimated as suggested by Weiss [14], using TDN = (CPd+NFCd+NDFapd+ [EEd×2.25]), where CPd, digestible crude protein; NDFapd, digestible neutral detergent fibre corrected to protein and ash; NFCd, digestible non-fibrous carbohydrate; EEd, digestible ether extract). The digestible energy (DE) was estimated as DE (Mcal/kg) = 0.04409×TDN (%). The conversion of DE to metabolizable energy (ME) was estimated as DE×0.82. The DM intake, ME intake, feed conversion (FC) and feed efficiency used in the results and discussion of the present study were obtained by Andrade et al [15].

After 66 days of feeding, the lambs were fasted for 16 hours, weighed to obtain body weight at slaughter (BWS) and then slaughtered by cerebral concussion followed by jugular and carotid venesection. Pre-harvest handling was in accordance with good animal welfare practices and our slaughtering procedures followed the Sanitary and Industrial Inspection Regulation for Animal Origin Products [16].

After obtaining BWS, bleeding, evisceration and head and limb removal were performed, and the carcass was weighed to obtain the hot carcass weight (HCW). The gastrointestinal tract was also weighed when it was full and then when it was empty, and it was weighed again to obtain the weight of the contents of the gastrointestinal tract and to calculate the empty body weight (EBW). The yields of the hot (HCY) and biological (BY) carcass were determined using the formulas HCY (%) = (HCW/BWS)×100 and BY (%) = (HCW/EBW)×100.

Subsequently, the carcasses were taken to the cold room with an average temperature of 4°C where they remained for 24 hours, and after this cooling period they were weighed to obtain the cold carcass weight (CCW). Cold carcass yield (CCY) and cooling losses (CL) were determined according to the equations CCY (%) = (CCW/BWS)×100 and CL (%) = (HCW−CCW/HCW)×100.

After the cooling period, morphometric measurements were performed on the carcasses. The carcass compactness index (CCI) and the leg compactness index (LCI) were also calculated using the following equations: CCI (kg/cm) = CCW/internal length carcass; and LCI (cm/cm) = hind width/leg length [17]. After which each carcass was divided sagittally. The left halves of the carcasses were sectioned in six anatomical regions that constitute meat cuts according to the methodology adapted from Cezar and Souza [17]: neck, shoulder, rib, saw, loin, and leg.

To obtain the *longissimus* muscle area in the left halves of the carcasses, a cut was performed between the 12th and 13th ribs to expose the *longissimus dorsi* muscle. This area was hatched on a transparent plastic sheet and was measured later using a digital planimeter (HAFF, Digiplan model; Pfronten, Baviera, Germany). In the same muscle, we measured subcutaneous fat thickness using callipers. The carcass pH was measured at 45 minutes and 24 hours post mortem in the *semimembranosus* muscle using a portable pH meter (Testo, model 205; Testo Instrument Co. LTD., Berlin, Germany).

### Statistical analysis

The experimental design was a randomized block with four treatments and ten replicates. The initial weight of the animals was the criterion for the formation of blocks according to the model:

$$Y_{ij}=\mu +T_{i}+b_{j}+e_{ij}$$

where *Y_ij_* = observed value of the dependent variable; *μ* = general mean; *T_i_* = treatment effect (i = 1 to 4); *b_j_* = effect of block *j* (j = 1 to 4) and *e_ij_* = experimental error. The variables studied were interpreted by analysis of variance and regression analysis, considering the level of 5% probability for the type I error, using general linear model and REG procedures for L linear and Q quadratic effect of the SAS software package [18].

## RESULTS

### Carcass characteristics

There was an increasing linear effect (p<0.05) on BWS with replacement of corn by CG (Table 3). The DM intake, ME intake (Mcal/kg of DM), EBW, HCW, and CCW presented a quadratic effect (p<0.05) (Table 3), with maximum values of 1,293.76 g/d, 3.27, 31.33, 18, 10, and 17.15 kg, estimated at CG levels of 10.9%, 9.8%, 10.83%, 11.78%, and 11.35%, respectively. The FC presented a quadratic effect (p<0.05), with an estimated 4.7 minimum conversion for the 10.5% level of replacement, while the feed efficiency was not influenced by treatments (Table 3).

There was no influence (p >0.05) of the corn replacement levels by CG on hot and cold carcass yields, BY, or CL % (Table 3). There was no effect (p>0.05) of the diets on the *longissimus* muscle area or subcutaneous fat thickness (Table 3). The initial pH (45 min) after slaughter was not influenced (p>0.05), but the final pH (24 h) presented quadratic behaviour (p< 0.05), with a minimum value of 5.28 that was estimated at the CG level of 12% (Table 3).

### Weights and yields of commercial cuts

The weight and yields of the meat cuts (neck, shoulder, ribs, saw, loin, and leg) were not influenced (p>0.05) by replacement (Table 4).

### Morphometric measurements

There was a quadratic effect (p<0.05) on the thoracic perimeter, with a maximum length of 71.63 cm, estimated at the CG level of 12.20% (Table 5). The other morphometric measurements were not influenced (p>0.05) (Table 5). The LCI was not influenced (p>0.05) by the replacement of corn with CG, but an increasing linear effect (p<0.05) was found for the CCI (Table 5).

## DISCUSSION

The observed behaviour for the EBW, HCW, and CCW variables may be related to DM intake and ME intake due the fact that glycerol from CG may have increased dietary energy efficiency by the microorganisms in the rumen and consequently favoured the synthesis of tissues in the body of the animal. The BWS values are between the intervals of studies on sheep of the same age that were fed CG in confinement and were slaughtered when they were between 25 and 38 kg [7,8,19,20]. In the present study the animals were slaughtered at an average of 35.21 kg and presented a linear behaviour that increased with the inclusion of CG in the diets, with a better performance of the animals as the amount of corn replaced by CG increased.

The values found for the CL % of the carcasses were within the range cited by Martins et al [21] which is between 1% and 7%. Lage et al [19] reported lower HCY (44.32%) and CCY (42.92%) values when they evaluated the effects of CG (36.2% glycerol). However, their CG inclusions were lower, mainly the glycerol content present in glicerin, reflecting the metabolism and utilization of glycerol by the animals. In addition, the carcass yield is directly affected by carcass weight. The performance presented (Table 3) by the animals in confinement was reflected in the HCW, which presented satisfactory values and influenced the significant performance in carcass yield.

The *longissimus* muscle area that we obtained can be considered satisfactory. Lage et al [19] evaluated the effects of inclusion of CG with 36.2% of glycerol in the diet of confined sheep slaughtered at a mean of 32.72 kg and found an average of 12.1 cm^2^, while Rego et al [8] and Carvalho et al [20] obtained much higher values of 13.66 and 14.44 cm^2^. According to Cezar and Souza [17] the determination of the *longissimus* muscle area measured as the *longissimus dorsi* muscle has traditionally been used as a good estimate of carcass musculature and is directly correlated with the muscle/bone relationship in the most important cuts of the carcass, which exerts an important influence on the classification of the carcass and the evaluation of the final price of the meat.

The absence of an influence of the diets on subcutaneous fat thickness could be related to the time of confinement, which was of 90 days for animals that were four months old. Consequently, slaughter occurred before the adipose tissue began its major deposition because according to Gerrard and Grant [22] adipose tissue develops last, after peak muscle growth. According to Osório et al [23] subcutaneous fat thickness is associated with several factors, including race, gender, diet, the duration of the feeding period and confinement. Therefore, it was not related to the energetic level of the diets, but to the chronology of body development, meaning the animals did not reach their maximum body development.

The behaviour observed for the final pH (24 h) may have occurred as a result of the increased ME intake with increasing CG levels. A hypothesis for this behaviour would be that the increase in muscle glycogen reserves, which were converted to lactic acid later, thereby reduced the final shell pH. Silva Sobrinho et al [24] found that the final pH value of sheep meat can range from 5.5 to 5.8. Therefore, the pH that we found at 24 hours was close to this range, indicating no pre-slaughter stress.

Regarding the commercial cuts, the leg was the cut of greater weight and, consequently, higher yield (Table 4). According to Silva Sobrinho [25] this becomes important because it is a region with greater muscularity and a greater yield of the edible part. In addition to the leg, the shoulder and the loin are the most valued commercial cuts of the carcass. Thus, when greater yields of these cuts are obtained, the carcass has a higher value. In this research, the three cuts in question comprised 60.08% of the yield. These results correspond to those reported by Furusho-Garcia et al [26] where the shoulder and leg represented more than 50% of the carcass, and these cuts are the best predictors of the total contents of the carcass tissues.

Body development occurs in the following sequence: bones, muscle and fat. The bone structure is almost fully developed, in dimension, in the first months of life of the animal, followed by muscle hypertrophy and finally the deposition of adipose tissue [22]. The morphometric results observed in this study demonstrate that the linear and circular measurements of the carcass did not vary statistically between the inclusion levels of CG in the diet.

The increasing linear effect observed on the CCI is related to the CCW, which also increased linearly with replacement. The values found are within the range of the indexes for sheep that are documented in the literature, indicating good deposition of muscle tissue per unit length. The higher the CCI, the greater the deposition of muscle tissue per unit area and the better the carcass will be evaluated. Thus, the replacement of corn by CG favours the deposition of muscle tissue.

## CONCLUSION

Crude glycerin can replace up to 18% of corn and favour the muscle tissue deposition. Therefore, it presents itself as an excellent alternative food, contributes to obtaining more valued carcasses and consequently, corresponds to the final value of commercialized meat.

## Figures and Tables

**Table 1:** Chemical composition of ingredients on a dry matter basis (g/kg of DM)

Item	Crude glycerin	Soybean meal	Ground corn	Tifton hay
Dry matter (g/kg fresh weight)	899	879	889	902
Organic matter	889.0	928.7	984.7	918.9
Ash	111	71.3	15.3	81.1
Crude protein	16.0	473	93.5	77.3
Ether extract	43.5	28.5	62.6	7.9
NDFap	-	165	160	735
NFC	-	237	686	122
Glycerol	805	-	-	-
Sodium	5.3	-	-	-
Methanol	38.2	-	-	-
Density (g/cm^3^)	0.97	-	-	-

DM, dry matter; NDFap, neutral detergent fibre corrected for ash and protein; NFC, non-fibrous carbohydrates.

**Table 2:** Proportion of ingredients and chemical composition of the experimental diets

Item	Levels of crude glycerin (%)			

	0	6	12	18
Ingredients (g/kg)				
Tifton hay	40.00	40.00	40.00	40.00
Ground corn	40.35	34.10	27.85	21.60
Soybean meal	17.00	17.00	17.00	17.00
Crude glycerin	0.00	6.00	12.00	18.00
Urea: AS1)	0.50	0.75	1.00	1.25
Mineral supply2)	1.50	1.50	1.50	1.50
Limestone calcitic	0.30	0.30	0.30	0.30
Bicalcium phosphate	0.35	0.35	0.35	0.35
Chemical composition (g/kg DM)				
Dry matter (g/kg fresh weight)	890	891	894	892
Organic matter	921.5	915.7	910.0	904.3
Crude protein	161.6	163.3	164.9	166.6
Ether extract	33.3	32.0	30.4	29.2
NDFap3)	386.2	376.3	366.3	356.4
Non-fiber carbohydrates	348.5	356.2	364.5	372.2
Total digestible nutrients4)	689	694	694	690

AS, ammonium sulphate; DM, dry matter; NDFap, neutral detergent fiber corrected for ash and protein.

1) 9 parts of urea and 1 part of ammonium sulphate (AS)

2) Assurance levels provided by the manufacturer: Calcium, 150 g; sulphur, 12 g; phosphorus, 65 g; magnesium-6.000 mg; sodium, 107 g; copper, 100 mg; cobalt, 175 mg; iron, 1,000 mg; fluorine, 650 mg; iodine, 175 mg; manganese, 1,440 mg; selenium, 27 mg; zinc, 6,000 mg.

3) Nine parts of urea and 1 part of ammonium sulfate (AS).

4) Estimated in the digestibility assay.

**Table 3:** Productive parameters and carcass characteristics of lambs fed with crude glycerin in replacement for corn

Item	Levels of crude glycerin (% DM)				SEM	p-value	
	
	0	6	12	18		L	Q
Productive parameters							
Dry matter (g/d) *	1,251.3	1,286.9	1,303.2	1,272.9	20.94	0.152	0.0431)
Metabolizable energy (Mcal/kg)*	3.12	3.23	3.27	3.17	10.67	0.426	0.0342)
Feed conversion*	5.5	5.0	4.8	5.1	0.073	0.345	0.0413)
Feed efficiency*	17.5	19.7	20.5	19.3	0.489	0.136	0.086
Carcass characteristics							
Body weight at slaughter (kg)	32.97	35.44	36.65	35.77	0.453	0.0144)	0.053
Empty body (kg)	28.62	30.45	31.62	30.00	0.355	0.080	0.0125)
Hot carcass weight (kg)	16.02	17.83	17.89	17.61	0.213	0.004	0.0046)
Cold carcass weight (kg)	15.43	16.94	17.04	16.72	0.203	0.022	0.0187)
Cooling losses (%)	5.08	4.96	5.25	5.06	0.119	0.324	0.284
Hot carcass yield (%)	50.56	50.45	49.14	49.28	0.440	0.209	0.896
Cold carcass yield (%)	46.84	47.93	46.57	46.78	0.373	0.659	0.565
Biological yield (%)	58.17	58.61	56.93	58.77	0.440	0.977	0.453
*Longissimus* muscle area (cm^2^)	11.47	11.51	11.94	11.08	0.298	0.791	0.471
Fat thickness (mm)	0.90	0.96	0.97	0.90	0.038	0.450	0.235
pH inicial	6.54	6.51	6.57	6.50	0030	0.786	0.723
pH ultimate	5.43	5.34	5.32	5.42	0.021	0.806	0.0318)

DM, dry matter; SEM, standard error of the mean; L, linear effect; Q, quadratic effect.

1) Ŷ = 1258.21+6.499X–0.297X^2^

2) Ŷ = 3.11+0.0294X–0.0015X^2^.

3) Ŷ = 5.24–0.089X+0.0048X^2^.

4) Ŷ = 32.93+0.579X.

5) Ŷ = 28.52+0.520X–0.024X^2^.

6) Ŷ = 16.09+0.3417X–0.0145X^2^.

7) Ŷ = 15.48+0.295X–0013X^2^.

8) Ŷ = 5.43–0.024X+0001X^2^.

* Andrade et al [15].

**Table 4:** Weight and yield of commercial cuts of lambs fed with crude glycerin in replacement for corn

Item	Levels of crude glycerin (% DM)				SEM	p-value	
	
	0	6	12	18		L	Q
Weight (kg)							
Neck	0.72	0.80	0.82	0.78	0.028	0.443	0.340
Shoulder	1.34	1.49	1.34	1.35	0.025	0.636	0.158
Ribs	1.48	1.55	1.62	1.56	0.029	0.216	0.274
Saw	0.75	0.93	0.90	0.87	0.032	0.251	0.110
Loin	0.72	0.81	0.78	0.79	0.018	0.221	0.273
Leg	2.54	2.75	2.57	2.70	0.046	0.487	0.645
Yield (%)							
Neck	9.59	9.53	10.20	9.73	0.318	0.723	0.760
Shoulder	17.77	17.92	16.73	16.79	0.262	0.085	0.928
Ribs	19.64	18.59	20.18	19.37	0.282	0.766	0.833
Saw	9.90	10.93	11.24	10.77	0.286	0.269	0.204
Loin	9.49	9.76	9.74	9.85	0.190	0.547	0.843
Leg	33.60	33.26	31.91	33.49	0.392	0.640	0.223

DM, dry matter; SEM, standard error of the mean; L, linear effect; Q, quadratic effect.

**Table 5:** Morphometric measurements of carcass (cm) of lambs fed with crude glycerin in replacement for corn

Item	Levels of crude glycerin (% DM)				SEM	p-value	
	
	0	6	12	18		L	Q
External carcass length	56.90	57.60	57.70	57.65	0.367	0.496	0.627
Internal carcass length	63.10	64.30	64.60	63.35	0.379	0.762	0.120
Thoracic width	21.45	22.10	22.50	22.50	0.226	0.089	0.479
Thoracic perimeter	67.45	70.70	71.50	70.75	0.159	0.001	0.0221)
Thoracic depth	24.95	25.20	25.40	25.20	0.467	0.524	0.500
Hind width	22.75	22.73	22.35	22.80	0.168	0.883	0.504
Hind perimeter	62.65	64.90	64.30	64.65	0.378	0.112	0.208
Leg length	41.65	40.90	42.30	42.20	0.190	0.062	0.364
Leg perimeter	39.70	39.95	38.70	39.40	0.297	0.433	0.712
CCI (kg/cm)	0.24	0.26	0.26	0.26	0.003	0.0102)	0.060
LCI (cm/cm)	0.55	0.56	0.53	0.54	0.005	0.279	0.880

DM, dry matter; SEM, standard error of the mean; L, linear effect; Q, quadratic effect; CCI, carcass compactness index; LCI, leg compactness index.

1) Ŷ = 67.49+0.6783–0.0278X^2^.

2) Ŷ = 0.24+0.003X.
